# Comparative Diagnostic Performance of Cone Beam Computed Tomography and Dual‐Energy X‐Ray Absorptiometry for Low Bone Density Assessment. A Systematic Review and Meta‐Analysis

**DOI:** 10.1002/cre2.70423

**Published:** 2026-08-01

**Authors:** Mohammad Alrashidi, Haitham Elbishari, May Aljanahi, Fatemeh Amir‐Rad, Jahanzeb Chaudhry, Momen Atieh

**Affiliations:** ^1^ Hamdan Bin Mohammed College of Dental Medicine Mohammed Bin Rashid University of Medicine and Health Sciences Dubai UAE; ^2^ School of Medicine, Dentistry and Biomedical Sciences Queen's University Belfast Belfast UK; ^3^ The Faculty of Biology, Medicine and Health The University of Manchester Manchester UK; ^4^ Faculty of Dentistry, Sir John Walsh Research Institute University of Otago Dunedin New Zealand; ^5^ School of Dentistry The University of Jordan Amman Jordan

**Keywords:** bone density, cone‐beam computed tomography, dental implants, diagnostic accuracy, osteopenia, osteoporosis

## Abstract

**Objective:**

This systematic review evaluated the diagnostic accuracy of CBCT for BMD assessment using DEXA as the reference standard and aimed to quantify its performance through meta‐analysis.

**Materials and Methods:**

This review included prospective and retrospective observational studies assessing CBCT's diagnostic performance in detecting low BMD, with DEXA as the reference. Searches in PubMed, Embase, and Scopus identified 13 eligible studies involving adult participants (≥ 18 years). Two reviewers independently extracted data and assessed study quality using the QUADAS‐2 tool. Meta‐analysis was conducted using random‐effects models.

**Results:**

Pooled sensitivity and specificity of CBCT for detecting low BMD were 0.58 (95% CI: 0.50–0.67) and 0.78 (95% CI: 0.69–0.86), respectively. The summary receiver operating characteristic (SROC) curve showed an area under the curve (AUC) of 0.73 (95% CI: 0.69–0.77), indicating moderate diagnostic accuracy. Subgroup analyses revealed heterogeneity influenced by sample size and anatomical site.

**Conclusion:**

CBCT demonstrates moderate diagnostic accuracy in detecting low BMD, with better specificity than sensitivity. While promising, standardization of CBCT protocols and larger studies are needed to confirm its role in clinical assessment.

**PROSPERO Registration Number:**

(CRD42024598958).

## 1 Introduction

Bone mineral density (BMD) assessment is essential for diagnosing osteoporosis and evaluating fracture risk (Johnell and Kanis 2006). Dual‐energy X‐ray absorptiometry (DEXA) remains the gold standard, categorizing bone health into normal, osteopenia, or osteoporosis based on T‐scores (Professor Kanis et al. 1994). Factors such as age, hormonal changes, and lifestyle choices significantly influence BMD, notably in postmenopausal women (Reginster and Burlet 2006). Advancements such as high‐resolution peripheral quantitative computed tomography (HR‐pQCT) provide detailed views of bone microarchitecture, improving fracture risk assessment (Boutroy et al. 2005). Regular monitoring of BMD is crucial for those diagnosed with osteopenia or osteoporosis to evaluate the effectiveness of treatment strategies (Cosman et al. 2014).

Traditional radiographs, while effective at identifying fractures, are insufficient for accurately measuring BMD or detecting early‐stage bone deterioration due to their inability to provide quantitative data on bone quality and structure (Grados et al. 2009). The advent of DEXA provided a more accurate and reliable method for BMD assessment, enabling early diagnosis of osteoporosis. However, DEXA's two‐dimensional representation of bone does not fully capture its three‐dimensional architecture, which remains a limitation (Kennel et al. 2020). Quantitative Computed Tomography (QCT) enables 3D visualization of bone structure, allowing volumetric assessment of both density and architecture. However, its clinical use remains limited due to higher radiation exposure and costs compared to DEXA (Gausden et al. 2017). More recently, HR‐pQCT has enhanced the ability to assess bone microarchitecture, offering key insights into bone quality and strength beyond BMD alone (Burt et al. 2023). Magnetic Resonance Imaging (MRI) also aids in evaluating bone health by visualizing marrow composition and detecting microfractures, though its routine use is limited by high cost and limited availability (Anderson et al. 2019). Additionally, evaluating Bone Turnover Markers (BTMs) in biological fluids is a promising approach to monitoring bone metabolism, offering a broader perspective on bone health when combined with imaging techniques. Nonetheless, the reliability of BTMs can be compromised by their inherent fluctuations and susceptibility to external influences (Vasikaran et al. 2011).

Cone‐Beam Computed Tomography (CBCT) is widely used in dentistry, providing high‐resolution 3D imaging with lower radiation than conventional CT. It aids in evaluating bone architecture and density, supporting precise implant planning (Shah 2017). Furthermore, its precision in forecasting implant dimensions and recognizing the necessity for bone grafting highlights its significance in preoperative planning (Deeb et al. 2017). Despite its benefits, using CBCT to assess BMD is still debated. Although it offers high dimensional accuracy similar to traditional CT, its reliance on gray values rather than direct density measurements limits its precision, especially for preoperative planning (Fahd and ElBeshlawy 2023). The reliability of CBCT in assessing bone density is still uncertain. Some studies report linear correlations with Hounsfield Units (HU), indicating its potential for low bone mass screening, while others point to inconsistent findings and emphasize the need for cautious interpretation (Poiana et al. 2023).

A major challenge in CBCT‐based bone density assessment is the lack of standardization across devices. Differences in gray values and imaging artifacts can compromise measurement accuracy and hinder reliable evaluation of bone quality (Sennerby et al. 2015). Variability among CBCT systems highlights the need for proper calibration or adjustment to ensure reliable bone density assessments and accurate implant planning (Fahd and ElBeshlawy 2023).

Considering such limitations, CBCT can nevertheless yield significant insights on patterns of bone structure. CBCT‐derived metrics, such as the Mandibular Osteoporosis Index (MOI), have demonstrated efficacy in differentiating osteoporosis from normal bone density. This feature facilitates the utilization of CBCT in evaluating osteoporosis risk and conducting preoperative assessments for dental implants by identifying individuals with an elevated risk of implant failure due to impaired bone quality (Poiana et al. 2023). CBCT has also shown significant correlation with microCT findings in in‐vitro evaluations of trabecular bone architecture, indicating that both imaging techniques provide unique benefits in evaluating bone quality for implant planning (Sennerby et al. 2015). This systematic review and meta‐analysis aimed to assess the diagnostic accuracy of CBCT in evaluating BMD, using DEXA as the reference standard.

## 2 Materials and Methods

The study was conducted in accordance with the guidelines of Preferred Reporting Items for Systematic Reviews and Meta‐Analyses of Diagnostic Test Accuracy Studies (PRISMA‐DTA) (McInnes et al. 2018). The outcomes were then reported using the PRISMA checklist (Page et al. 2021).

The eligibility criteria attempted to systematically answer the focused question using the Population, Intervention, Comparison, Outcome (PICO) framework (Miller and Forrest 2001) identified as follows:

**Population (P)**: adult patients who had DEXA and CBCT assessment for low BMD

**Intervention (I):** CBCT

**Comparison (C):** DEXA

**Outcome (O):** Diagnostic accuracy (e.g., sensitivity, specificity) for detecting low BMD (Table 1)

**Table 1:** Search strategy based on the PICO framework.

Focus question: Comparative diagnostic performance of CBCT and DEXA for BMD assessment	
Population	Adults (≥ 18 years), both males and females
Intervention	CBCT
Comparison	DEXA
Outcome	Diagnostic accuracy (sensitivity, specificity)
Limits	English language, humans, clinical studies

The review was registered with the National Institute for Health Research (NHR) under the PROSPERO ID CRD42024598958. Ethical approval was not required.

### 2.1 Inclusion Criteria

Prospective or retrospective observational cross‐sectional studies evaluating the diagnostic performance of CBCT compared to DEXA (Table 2).

**Table 2:** Inclusion and exclusion criteria for study selection.

Criteria	Inclusion criteria	Exclusion criteria
Study type	Original observational studies including cross‐sectional, cohort, or case‐control designs	Case reports, case series, narrative reviews, systematic reviews, editorials, conference abstracts, animal or in vitro studies
Participants	Human adults (≥ 18 years), both males and females, undergoing both CBCT and DEXA for bone mineral density assessment	Studies involving pediatric populations or non‐human subjects
Intervention	Use of CBCT for the evaluation or prediction of bone mineral density	Studies using imaging modalities other than CBCT
Comparison	Comparison with DEXA as the reference standard for assessing bone mineral density	Studies without DEXA comparison or using other reference standards
Outcomes	Studies reporting diagnostic performance metrics (e.g., sensitivity, specificity, PPV, NPV, or TP, TN, FP, FN data)	Studies not reporting sufficient diagnostic accuracy outcomes or not allowing derivation of such metrics
Language	Studies in English	Studies not in English Language

Abbreviations: FN, false negative; FP, false positive; NPV, negative predictive value; PPV, positive predictive value; TN, true negative; TP, true positive.

### 2.2 Exclusion Criteria

In‐vitro studies, case reports, and studies lacking quantitative information about the outcomes, lacking a direct comparison using DEXA, or conducted in languages other than English (Table 2).

### 2.3 Type of Participants

Adult males and females (≥ 18 years of age). All participants underwent CBCT for low BMD assessment.

### 2.4 Type of Intervention

CBCT was evaluated as the index test for assessing low BMD, with DEXA serving as the reference standard.

### 2.5 Outcomes of Interest

The primary outcomes were measures of diagnostic accuracy, including sensitivity and specificity of CBCT for detecting low BMD, using DEXA as the reference standard. Secondary outcomes included positive and negative predictive values, likelihood ratios, diagnostic odds ratios, and area under the receiver operating characteristic (ROC) curve.

### 2.6 Search Strategy and Study Selection

The electronic databases PubMed, Scopus, Web of Science, Ovid MEDLINE, and Ovid Embase were systematically searched from January 1965 to February 2025 to identify relevant studies. The detailed search strategies for each database, including all keywords used, are presented in Table 3. The search was conducted by two independent reviewers (M.R. and M.J.) independently and in duplicate; examined the retrieved studies based on the title, abstract, and keywords. Duplicates were removed, and irrelevant studies were excluded. The full text of the remaining studies was reviewed. Any disagreements were resolved by discussion to reach a consensus or by consultation with a third reviewer (H.E.). In the event of duplicate studies, the most relevant studies with sufficient information and the longest follow‐up period were selected.

**Table 3:** Databases and literature search terms.

Database (January 1965–February 2025)	Search strategy
PubMed	(((CBCT OR “cone beam computed tomography” OR “cone beam” OR CBVT OR conebeamvolumetomography)) AND (“bone mineral density” OR “bone density” OR “bone quality” OR osteoporosis OR osteopenia OR “mandibular bone density” OR “jaw bone density” OR “mandibular cortical index” OR “radiomorphometric index”)) AND (“dual energy X‐ray absorptiometry” OR DXA OR DEXA OR densitometry OR densitometric OR “DXA scan”))
Scopus	TITLE‐ABS‐KEY((“cone beam computed tomography” OR CBCT OR “cone beam” OR CBVT OR conebeamvolumetomography) AND (“bone mineral density” OR “bone density” OR “bone quality” OR osteoporosis OR osteopenia OR “mandibular bone density” OR “jaw bone density” OR “mandibular cortical index” OR “radiomorphometric index”) AND (“dual energy X‐ray absorptiometry” OR DXA OR DEXA OR densitometry OR densitometric OR “DXA scan”)
Ovid MEDLINE	(CBCT OR “cone beam computed tomography” OR “cone beam” OR cone‐beam OR CBVT OR conebeamvolumetomography).mp. AND (bone mineral density OR bone density OR osteoporosis OR osteopenia OR mandibular cortical index OR radiomorphometric index).mp. AND (dual energy x ray absorptiometry OR DXA OR DEXA OR densitometry OR densitometric OR “DXA scan”).mp.
Web of Science	(“cone beam computed tomography” OR CBCT OR “cone beam” OR “cone‐beam” OR CBVT OR conebeamvolumetomography) AND (“bone mineral density” OR “bone density” OR osteoporosis OR osteopenia OR “bone quality” OR “mandibular cortical index” OR “radiomorphometric index”) AND (“dual energy x ray absorptiometry” OR DXA OR DEXA OR densitometry OR densitometric OR “DXA scan”)
Embase	(cone beam computed tomography OR CBCT OR CBVT OR conebeamvolumetomography).mp. AND (bone mineral density OR bone density OR osteoporosis OR osteopenia OR bone quality OR mandibular cortical index OR radiomorphometric index).mp. AND (dual energy x ray absorptiometry OR DXA OR DEXA OR densitometry OR densitometric OR “DXA scan”).mp.

### 2.7 Data Extraction

The following information was extracted from the studies:

i. **Study characteristics:** title, authors’ names, study location, year of publication.
ii. **Participants:** demographic characteristics, number of participants in both groups.
iii. **Interventions:** CBCT assessment of low BMD, including the site assessed and method used.
iv. **Comparison**: DEXA assessment of low BMD, including the anatomical site and method used.
v. **Outcomes**: Diagnostic performance measures including True Positive (TP), True Negative (TN), False Positive (FP), False Negative (FN), Sensitivity, Specificity, Positive Predictive Value (PPV), and Negative Predictive Value (NPV).

### 2.8 Quality Assessment

The methodological quality and risk of bias of the included studies were assessed using the QUADAS‐2 tool (Quality Assessment of Diagnostic Accuracy Studies‐2). This tool evaluates four key domains: Patient Selection, Index Test, Reference Standard, and Flow and Timing. Each domain was assessed for risk of bias, and the first three domains were also evaluated for concerns regarding applicability. Two independent reviewers (M.A. and M.J.) conducted the risk of bias assessment, and disagreements were resolved through discussion or consultation with a third reviewer (H.E.). The assessment covered the patient selection index test and reference standard flow and timing.

For each domain, the risk of bias was categorized as low, high, or unclear, with justifications based on study characteristics. Applicability concerns were rated as low, high, or unclear based on alignment with the review question.

### 2.9 Data Synthesis

The meta‐analysis was conducted using Stata version 18.5 (StataCorp, College Station, TX, USA). A bivariate random‐effects model was used to calculate the pooled estimates of sensitivity, specificity, and the summary receiver operating characteristic (SROC) curve with the area under the curve (AUC). Where available, 2 × 2 tables were extracted to allow calculation of diagnostic accuracy metrics.

Between‐study heterogeneity was assessed using Cochran's Q test and quantified by the *I* ^2^ statistic, with values > 50% indicating substantial heterogeneity. In addition, positive and negative likelihood ratios (LR^+^, LR^−^), positive predictive value (PPV), and negative predictive value (NPV) were calculated where applicable.

Subgroup analyses and univariate meta‐regression were performed to explore potential sources of heterogeneity, specifically assessing the influence of sample size (≥ 60 vs. < 60) and the anatomical site of CBCT assessment (e.g., cervical vertebra vs. mandible).

The Fagan nomogram was used to evaluate the clinical utility of CBCT by calculating post‐test probabilities based on pre‐test probabilities and likelihood ratios. Potential publication bias was assessed using Deeks’ funnel plot asymmetry test, with a *p*‐value < 0.05 indicating significant asymmetry.

## 3 Results

### 3.1 Characteristics of Study Design

**Figure 1:**
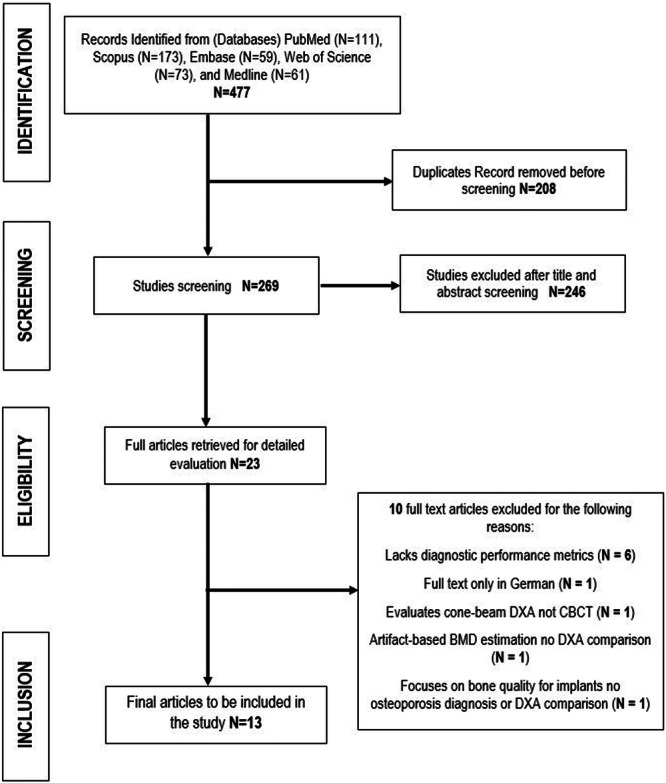
PRISMA flow diagram of study selection.

A comprehensive literature search yielded 477 records from electronic databases. After removing one duplicate, a total of 269 studies were screened based on title and abstract independently and in duplicate by two reviewers (M.A. and M.J.). Subsequently, 23 studies were eligible for full text review after which 10 studies were excluded and 13 studies were included in the systematic review (Barngkgei et al. 2014, 2016, 2015; Barra et al. 2022, 2021; Carvalho et al. 2022; de Castro et al. 2020; Esmaeli et al. 2017; Güngör et al. 2016; Kato et al. 2019; Shokri et al. 2019; Slaidina et al. 2022; Slaidina et al. 2023) (Figure 1). The 10 studies excluded after full‐text review, together with the specific reasons for exclusion, are summarized in Table S1.

### 3.2 Study Characteristics

The systematic review included 13 studies that evaluated the diagnostic accuracy of CBCT compared to DEXA for low bone density assessment. The studies varied in design but predominantly followed a cross‐sectional methodology. They were conducted at university hospitals (e.g., Barngkgei et al. 2014, 2015 [Damascus University]; Güngör et al. 2016 [Zirve and Dicle Universities]; Esmaeli et al. 2017 [Tabriz University of Medical Sciences and Imam Reza Hospital]), research centers (e.g., Barra et al. 2022, 2021 [Universidade de Brasília]; Carvalho et al. 2022 [also in collaboration with KU Leuven and Karolinska Institutet]), and dental institutions (e.g., Kato et al. 2019 [São Paulo State University]; Shokri et al. 2019 [Hamadan University of Medical Sciences]; de Castro et al. 2020 [University of Brasília]; Slaidina et al. 2022, 2023 [Riga Stradins University]), with a primary focus on assessing bone mineral density (BMD) in the mandible, cervical vertebrae, and lumbar spine.

The sample size was calculated in eight studies (e.g., Barngkgei et al. 2014, 2016, 2015; Esmaeli et al. 2017; Güngör et al. 2016; Shokri et al. 2019; Slaidina et al. 2022, 2023). Most studies classified subjects into normal, osteopenic, and osteoporosis groups based on DEXA‐derived T‐scores. CBCT‐based assessments varied in methodology, where gray value analysis (Barngkgei et al. 2014, 2015; Esmaeli et al. 2017; Güngör et al. 2016; Shokri et al. 2019; Slaidina et al. 2022, 2023), radiomorphometric indices (e.g., Barngkgei et al. 2016; Barra et al. 2022, 2021; de Castro et al. 2020; Güngör et al. 2016; Kato et al. 2019), and fractal dimension analysis (e.g., Carvalho et al. 2022; Güngör et al. 2016) were used.

Bone density was evaluated in different anatomical sites, some studies used cervical vertebrae (Barngkgei et al. 2015; Esmaeli et al. 2017; Slaidina et al. 2022) others used jaw bones (Barngkgei et al. 2014, 2016; Barra et al. 2022, 2021; Carvalho et al. 2022; de Castro et al. 2020; Güngör et al. 2016; Kato et al. 2019; Shokri et al. 2019; Slaidina et al. 2023). The characteristics of the included studies are presented in Table 4. Detailed summaries of imaging parameters, methodological features, and the diagnostic accuracy performance of CBCT in comparison with DXA are reported in Tables S2 and S3.

**Table 4:** The characteristics of included studies. Presents key diagnostic metrics (TP, FP, TN, FN) for each included study, along with information on study design, CBCT and DEXA methodology, anatomical sites assessed, and evaluation methods.

Study	Type of study	DEXA						CBCT						
		Total	Normal	Osteopenia	Osteoporotic	Site	Method	Total	Site	Method	TP	FN	TN	FP
Slaidina et al. (2022)	Cross‐sectional	127	39	57	31	Lumbar spine L2‐L4	T‐Score	127	Axial Second cervical vertebrae	Gray value	40	48	34	5
									Sagittal second cervical vertebrae		54	34	27	12
								126	Axial Third Cervical vertebrae		39	48	34	5
									Sagittal third cervical vertebrae		55	32	23	16
						Femural necks								
Slaidina et al. (2023)	Cross‐sectional	127	39	57	31	Lumbar spine L2‐L4	T‐Score	127	Lateral incisor CM	Gray value	87	1	39	0
									Lateral incisor T basal		72	16	31	8
									Lateral incisor T alveolar		72	16	31	8
									First premolar CM		87	1	39	0
									First premolar T basal		64	24	29	10
									First premolar T alveolar		64	24	29	10
									First molar CM		88	0	39	0
									First molar T basal		45	43	19	20
						Femural necks			First molar T alveolar		45	43	19	20
Barngkgei et al. (2014)	Cross‐sectional	38	10	15	13	Lumbar vertebrae L1–L4	T‐Score	38	Mandibular body slice	Gray value	13	15	9	1
		38	17	11	10	Femural neck	T‐Score	38	Mandibular body slice		10	10	15	2
Barngkgei et al. (2015)	Cross‐sectional	38	10	15	13	Lumbar vertebrae L1–L4	T‐Score	38	Right C1 vertebral GV	Gray value	26	2	9	1
									Left C1 vertebral GV		26	2	8	2
									C2 vertebral GV		25	3	9	1
									C2‐Dens GV		20	8	9	1
		38	17	11	10	Femural neck	T‐Score	38	Right C1 vertebral GV		17	4	12	5
									Left C1 vertebral GV		13	8	16	1
									C2 vertebral GV		15	6	14	3
									C2‐Dens GV		16	5	13	4
Farzad Esmaeli et al. (2017)	Cross‐sectional	108	50	36	22	Lumber spin	T‐Score	108	Left lateral mass of C1	Gray value	50	8	50	0
									Right lateral mass of C1		53	5	50	0
									Body of C2		54	4	50	0
									Dens of C2		41	17	34	16
Shokri et al. (2019)	Cross‐sectional	61	27	24	10	Lumber spin	T‐Score	61	Cancellous plus cortical bone at the site of mandibular incisors	Gray value	21	13	12	15
									Cancellous plus cortical bone at the site of mandibular premolars		19	15	12	15
									Cancellous plus cortical bone at the mandibular retromolar region		21	13	14	13
									Cancellous plus cortical bone at the site of maxillary incisors		16	18	14	13
									Cancellous plus cortical bone at the site of maxillary premolars		18	16	13	14
									Cancellous plus cortical bone at the site of maxillary tuberosity		20	14	16	11
Barra et al. (2022)	Cross‐sectional	40	8	32		Lumbar spine (L1‐L4)	T‐score	40	Posterior (P)	Radiomorphometric indices	11	21	7	1
									Molar (M)		12	20	7	1
									Anterior (A)		15	17	6	2
									Symphysis (S)		27	5	3	5
									Computed Tomography Mandibular Index (CTMI)		3	29	5	3
									Computed Tomography Index (Inferior) [CTI (I)]		23	9	4	4
						Proximal femur (neck and total)			Computed Tomography Index (Superior) [CTI (S)]		18	14	5	3
Barra et al. (2021)	Cross‐sectional	48	16	32		Lumbar spine (L1–L4)	T‐score	48	Molar (M) index	Radiomorphometric indices	24	8	11	5
									Symphysis (S) index		16	16	14	2
						Proximal Femur (neck and total)			Posterior (P) index		24	8	10	6
Camila Nao Kato et al. (2019)	Cross‐sectional	54	16	38		Femoral neck	T‐score	54	Panoramic reconstruction of the CBCT (5 mm slice thickness)	Radiomorphometric indices	24	14	7	9
						Lumbar spine (L1–L4)			Panoramic reconstruction of the CBCT (15 mm slice thickness)		19	19	8	8
									Panoramic reconstruction of the CBCT (25 mm slice thickness)		20	18	10	6
Barngkgei et al. (2016)	Cross‐sectional	38	17	21		Femoral neck	T‐score	38	Trabecular thickness (Tb.Th) of Dens	Radiomorphometric indices	13	8	15	2
									Maximum Tb.Th of dens (mm)		4	17	16	1
		38	10	28		Lumbar spin		38	Trabecular thickness (Tb.Th) of Dens		17	11	8	2
									Maximum Tb.Th of dens (mm)		11	17	9	1
de Castro et al. (2020)	Cross‐sectional	103	52		51	Lumbar spin	T‐score	103	3D MOI CQ	Radiomorphometric indices	28	23	48	4
						Hip			3D MOI PR (2.75 mm)		40	11	35	17
									3D MOI CS (2.75 mm)		39	12	36	16
Carvalho et al. (2022)	Cross‐ sectional	103	52		51	Lumbar spine (L1‐L4)	T‐score	103	Mandibular (ROI‐m)	Fractal dimension	28	23	37	15
						Total hip								
						Femoral neck								
Enes Güngör et al. (2016)	Cross‐sectional	90	31	33	26	Lumbar spine (L1–L3)	T‐score	90	Right maxilla	Fractal dimension	54	5	29	2
									Left maxilla		54	5	30	1
									Right condyle		49	10	27	4
									Left condyle		53	6	28	3
								90	CTMI	Radiomorphometric indices	55	4	26	5
									CTI (I)		56	3	28	3
									CTI (S)		56	3	28	3
								90	Right maxilla	Gray value	43	16	28	3
									Left maxilla		47	12	26	5
									Right mandible		50	9	26	5
									Left mandible		54	5	25	6
									Right condyle		18	41	14	17
						Femoral head			Left condyle		43	16	26	5

*Note:* A, anterior; CTI, (I)cortical thickness index (inferior); CTI (S), cortical thickness index (superior); CTMI, cortical thickness mental index; FN, false negative; FP, false positive; M, molar; NPV, negative predictive value; P, posterior; PM, pre‐molar; PPV, positive predictive value; S, symphysis; TN, true negative; TP, true positive.

### 3.3 Risk of Bias

A total of 13 studies were evaluated using the QUADAS‐2 tool. Across the included studies, the risk of bias related to patient selection was frequently rated as high. This was largely attributed to the use of case‐control designs and narrowly defined inclusion criteria, such as limiting participants to postmenopausal women. All studies employed DEXA as the reference standard, which is a standard test for diagnosing osteoporosis. In nearly all cases, the reference standard was consistently applied and interpreted independently of the index test, resulting in a low risk of bias in this domain.

The index test domain commonly showed a high risk of bias across all included studies due to limited reporting on whether CBCT examiners were blinded to the DEXA results. Only four studies clearly stated examiner blinding (Barra et al. 2021; de Castro et al. 2020; Kato et al. 2019), while the remaining provided insufficient information. None of the studies pre‐specified thresholds for CBCT‐derived measures, raising concerns about potential bias from data‐driven threshold determination.

For flow and timing, the majority of studies administered both the index and reference tests to all participants within an acceptable timeframe (typically within 3 months), and included all patients in the final analyses, yielding a low risk of bias in this area.

**Figure 2:**
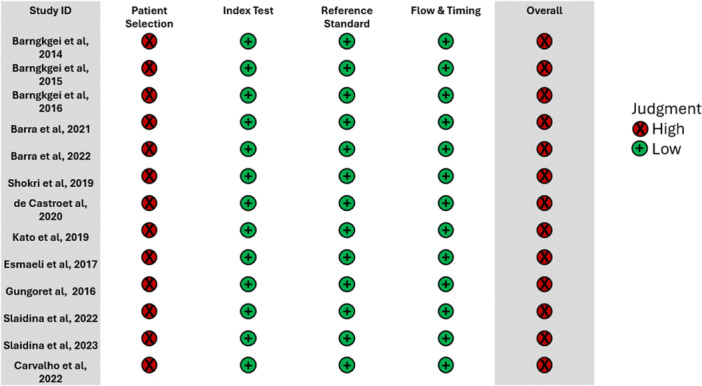
QUADAS‐2 assessment of risk of bias across included studies.

With respect to applicability, all studies demonstrated low concern across domains, as both the CBCT‐derived bone measures and the DEXA‐based definitions of osteoporosis were well aligned with the review question (Table 5; Figure 2).

**Table 5:** Risk of bias assessment using QUADAS‐2 tool.

Study	Patient selection	Index test	Reference standard	Flow and timing	Applicability: Patient selection	Applicability: Index test	Applicability: Reference standard
Barngkgei et al. (2014)	H	L	L	L	L	L	L
Barngkgei et al. (2015)	H	L	L	L	L	L	L
Barngkgei et al. (2016)	H	L	L	L	L	L	L
Barra et al. (2021)	H	L	L	L	L	L	L
Barra et al. (2022)	H	L	L	L	L	L	L
Shokri et al. (2019)	H	L	L	L	L	L	L
de Castro et al. (2020)	H	L	L	L	L	L	L
Kato et al. (2019)	H	L	L	L	L	L	L
Esmaeli et al. (2017)	H	L	L	L	L	L	L
Güngör et al. (2016)	H	L	L	L	L	L	L
Slaidina et al. (2022)	H	L	L	L	L	L	L
Slaidina et al. (2023)	H	L	L	L	L	L	L
Carvalho et al. (2022)	H	L	L	L	L	L	L

*Note:* Low (L) and high (H).

### 3.4 Diagnostic Accuracy Results

The diagnostic performance of CBCT for low bone density assessment was evaluated in the 13 included studies, with analysis of sensitivity, specificity, positive predictive value (PPV), and negative predictive value (NPV).

### 3.5 Sensitivity and Specificity Analysis

The forest plots of individual and pooled sensitivity and specificity values demonstrated variability across studies. The pooled sensitivity of CBCT for detecting low bone density was 0.58 (95% CI: 0.50–0.67), while the pooled specificity was 0.78 (95% CI: 0.69–0.86). The results indicate that CBCT exhibits moderate sensitivity but high specificity, suggesting that it is more reliable in ruling out false positives rather than detecting all true cases of low bone density.

**Figure 3:**
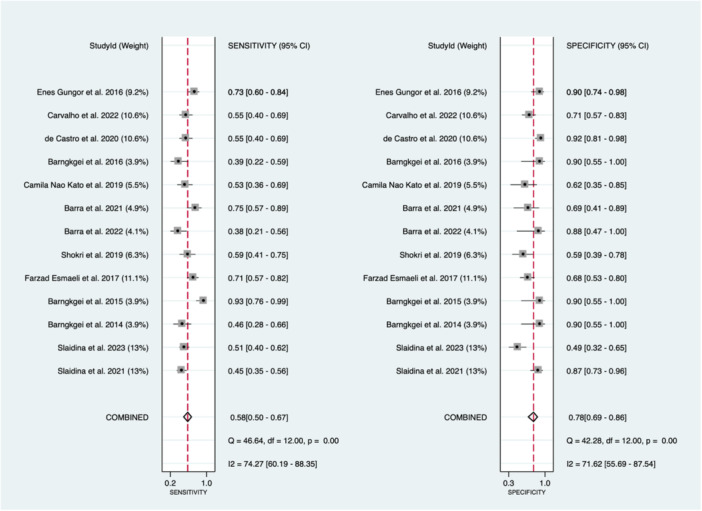
Forest plot of sensitivity and specificity estimates.

The heterogeneity analysis (*I* ^2^ values) for sensitivity and specificity was 74.27% and 71.62%, respectively, indicating a moderate to high degree of variability between studies. This suggests that differences in CBCT protocols, anatomical sites, and study populations may have influenced the results (Figure 3).

### 3.6 Summary Receiver Operating Characteristic (SROC) Curve

**Figure 4:**
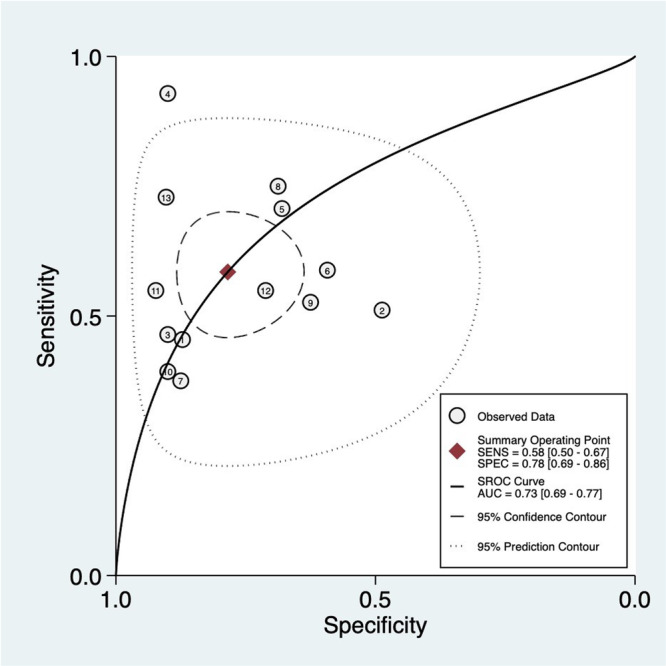
Summary receiver operating characteristic (SROC) curve.

The SROC curve analysis demonstrated an area under the curve (AUC) of 0.73 (95% CI: 0.69–0.77), which indicates moderate diagnostic accuracy. The positioning of individual study data points showed a concentration around the summary operating point, reinforcing the findings of moderate sensitivity and high specificity (Figure 4).

### 3.7 Likelihood Ratios (Fagan's Nomogram)

**Figure 5:**
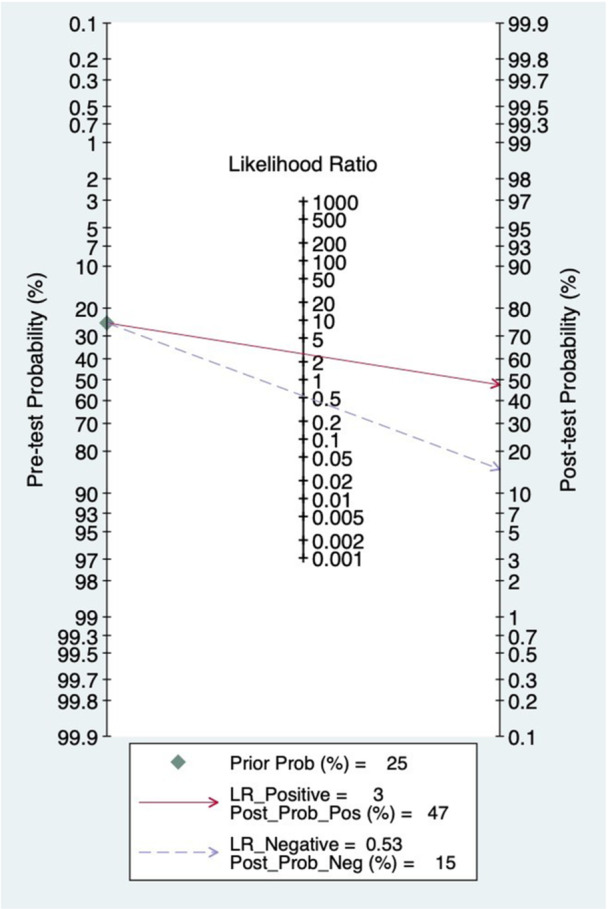
Fagan's nomogram for evaluating post‐test probabilities.

The Fagan nomogram analysis demonstrated that with a pre‐test probability of 25%, a positive likelihood ratio (LR + ) of 3 increased the post‐test probability to 47%, meaning CBCT improves the likelihood of confirming low bone density when positive. However, the negative likelihood ratio (LR‐) of 0.53 reduced the post‐test probability to 15%, suggesting a weaker ability to rule out low bone density when CBCT results are negative (Figure 5).

### 3.8 Publication Bias (Funnel Plot Analysis)

**Figure 6:**
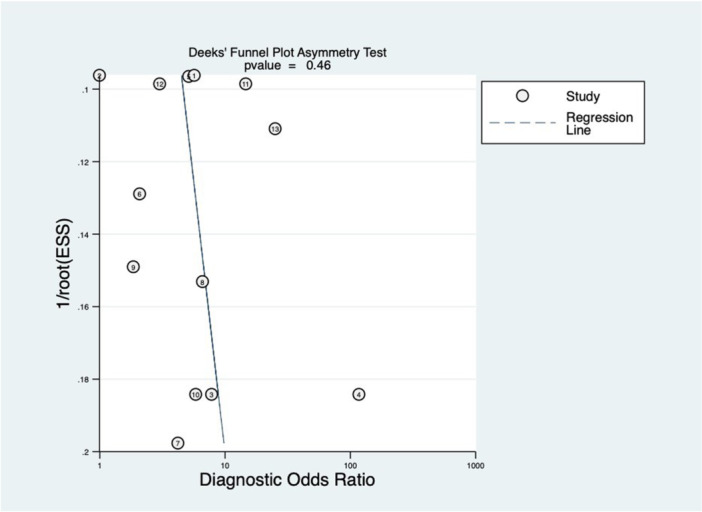
Funnel plot for publication bias assessment.

The Deeks’ funnel plot asymmetry test yielded a *p*‐value of 0.46, indicating that no significant publication bias was detected among the included studies. This strengthens the reliability of the meta‐analysis findings (Figure 6).

### 3.9 Subgroup Analysis and Meta‐Regression

**Figure 7:**
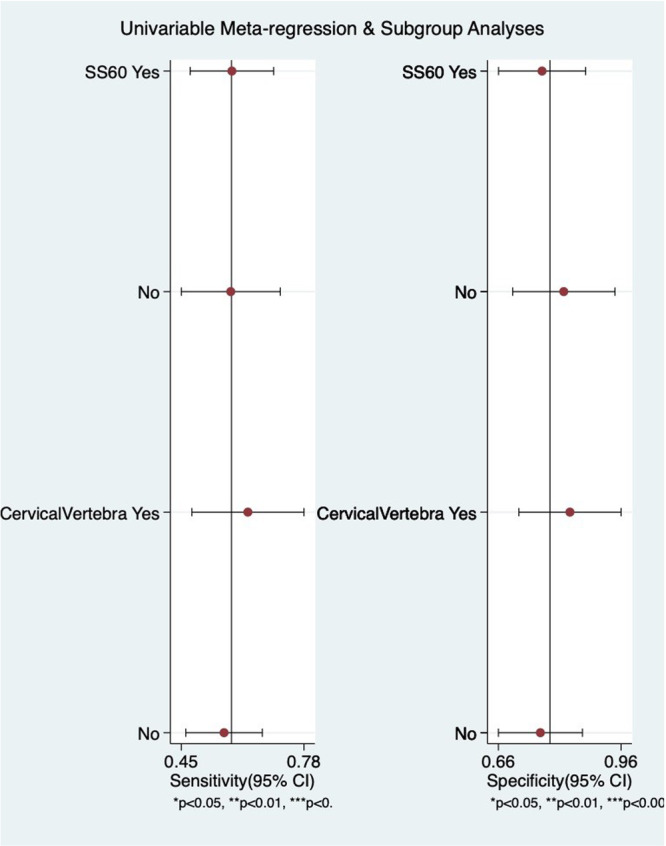
Subgroup analysis and univariable meta‐regression.

Meta‐regression analysis was conducted to explore potential sources of heterogeneity in CBCT diagnostic accuracy. Among the examined study‐level covariates, only subgrouping by sample size (≥ 60 participants) and anatomical site (cervical vertebrae vs. other sites) showed statistically significant associations with pooled sensitivity and specificity (*p* < 0.05) (Figure 7).

## 4 Discussion

This review evaluated the diagnostic performance of CBCT in comparison to the reference standard DEXA for identifying individuals with low BMD. These findings indicate that CBCT demonstrates a moderate level of diagnostic accuracy, with a tendency toward higher specificity than sensitivity. This pattern indicates that CBCT may be more effective at confirming low BMD when present, but less reliable in excluding the condition when CBCT findings appear normal.

The review focused on the diagnostic ability of CBCT to correctly classify individuals with and without low BMD. The results point to a reasonable capacity to avoid false‐positive diagnoses, although the likelihood of missing true cases remains a concern. This limits the utility of CBCT as a standalone screening tool but suggests potential value in confirmatory settings or when DEXA is not readily available.

Our findings also help to clarify the distinction between correlation‐based studies and diagnostic accuracy assessments. While previous research such as the systematic review and meta‐analysis by Francisco et al. (2023) has demonstrated a moderate to strong correlation between CBCT gray values and DEXA‐derived BMD scores, correlation alone does not inform clinical decision making in terms of classification accuracy. In contrast, diagnostic accuracy metrics such as sensitivity and specificity provide direct insight into how reliably a test can distinguish between affected and unaffected individuals, information that is crucial for clinical application.

The agreement between our findings and those of previous reviews is notable, particularly regarding the anatomical regions that appear most predictive. Both this review and the study by Francisco et al. (2023) identified the cervical vertebrae and mandibular regions as sites where CBCT performs relatively well. Our subgroup analyses further indicated that anatomical location was a significant source of variability, highlighting the importance of consistent landmark selection. This underlines a broader need for standardization in image acquisition and interpretation protocols, which could improve diagnostic consistency and comparability across future studies.

The findings of this review align with Poiana et al. (2023), who emphasized the potential of CBCT in identifying low bone mass, especially using mandibular and cervical vertebrae indices. While Poiana et al. conducted a qualitative synthesis of 16 studies, the current study presents a quantitative meta‐analysis, revealing moderate diagnostic accuracy. This research adds to the prior findings by integrating diagnostic performance metrics, examining heterogeneity, and affirming the role of CBCT as a supplementary tool in clinical contexts.

Compared to the systematic review by Guerra et al. (2017), key differences emerge in terms of both scope and analytical depth. Guerra et al. reviewed six studies and focused primarily on qualitative and radiomorphometric indices derived from CBCT, including mandibular cortical width and CT cortical index classifications. Although Guerra et al. (2017) concluded that CBCT‐derived measurements could distinguish osteoporotic individuals from control groups, the study did not conduct a meta‐analysis or report diagnostic accuracy measures such as sensitivity or specificity. Moreover, the review highlighted significant limitations in the existing body of literature, namely low sample sizes, methodological heterogeneity, and lack of standardized CBCT protocols. These findings reflect the earlier stage of research at the time, and the emphasis on the need for future studies is directly addressed by the larger sample and quantitative synthesis in the present review.

Variability in diagnostic accuracy across studies may partly be attributed to differences in the anatomical sites assessed. For instance, studies evaluating cervical vertebrae (e.g., Barngkgei et al. 2015; Esmaeli et al. 2017; Slaidina et al. 2022) reported relatively consistent values, whereas other studies focusing on mandibular sites showed wider variability. Additionally, CBCT protocol differences, including voxel size, machine type, software calibration, and region of interest selection, likely contributed to heterogeneity.

Meta‐regression identified sample size (≥ 60 vs. < 60) and anatomical site (cervical vertebrae vs. jaw) as statistically significant contributors to heterogeneity in pooled sensitivity and specificity; however, the magnitude of these effects was modest, and substantial residual heterogeneity persisted, as reflected by *I* ^2^ values of 74.3% for sensitivity and 71.6% for specificity. Although most included studies reported CBCT device models and imaging settings, variability in these parameters limited their inclusion in quantitative meta‐regression. Notably, none of the included studies reported pre‐specified diagnostic thresholds for CBCT, and examiner blinding was inconsistently described, factors that may have contributed to residual heterogeneity and influenced diagnostic performance estimates. The Fagan nomogram analysis supported the clinical applicability of CBCT in opportunistic screening scenarios. When applied to a population with a pre‐test probability of low BMD at 25%, a positive CBCT result increased the post‐test probability to approximately 47%, whereas a negative result reduced it to 15%. These findings indicate that CBCT can moderately enhance diagnostic confidence in identifying low BMD. However, the relatively low sensitivity of CBCT limits its ability to reliably rule out cases, which may constrain its role in exclusion‐based decision‐making or stand‐alone screening. Several confounding factors affecting bone mineral density, including body mass index, smoking status, and menopausal duration, were inconsistently reported across the included studies. Although some studies addressed these factors through exclusion criteria, they were not analytically adjusted for, which may have contributed to residual heterogeneity and bias in CBCT diagnostic performance estimates

This review represented the first meta‐analysis to comprehensively evaluate the diagnostic accuracy of CBCT in comparison to DEXA for detecting low BMD. The strengths of this review include strict adherence to PRISMA‐DTA reporting standards, use of the QUADAS‐2 tool to evaluate risk of bias, prospective registration in PROSPERO, and the application of subgroup and meta‐regression analyses to explore sources of heterogeneity. Another advantage is the inclusion of 13 studies encompassing various geographic regions and CBCT technologies, enhancing the generalizability and validity of the findings.

Despite the strengths, there were some limitations such as a lack of CBCT standardization across machines and imaging protocols, as well as high heterogeneity due to technique and anatomical variability. Most included studies were cross‐sectional, which limits causal and prognostic inferences. Furthermore, reporting on important confounding variables such as smoking status, body mass index, or concurrent medications was often insufficient. It is also worth noting that none of the studies included longitudinal outcomes such as fracture incidence or implant failure, which would provide more clinically relevant endpoints.

While not without limitations, CBCT holds potential as a supplementary tool for low BMD detection, especially in dental and maxillofacial settings where CBCT imaging is routinely conducted for other indications. Its high specificity supports its use in screening or triaging patients for further investigation via DEXA. In prosthodontic treatment planning, particularly for implant‐supported restorations, CBCT‐derived bone quality assessments may help identify patients at risk of compromised osseointegration.

## 5 Conclusion

Within the limitations of this review, CBCT demonstrated moderate diagnostic accuracy for detecting low BMD, with higher specificity than sensitivity. This meta‐analysis offers a more comprehensive evaluation than previous reviews by including more studies and reporting pooled diagnostic metrics. While CBCT is not a substitute for DEXA, it shows promise as an adjunctive screening tool in dental settings, especially where DEXA is unavailable. These findings support incorporating BMD risk assessment into routine dental imaging, enabling earlier identification of systemic bone health issues. However, widespread adoption will require standardized imaging protocols, calibration methods, and high‐quality research.

## Author Contributions

**Mohammad Alrashidi:** methodology, data curation, investigation, writing – original draft. **Haitham Elbishari:** conceptualization, methodology, validation, supervision, visualization, project administration, writing – review and editing. **May Aljanahi:** methodology, investigation, writing – original draft. **Jahanzeb Chaudhry:** methodology, supervision, writing – original draft. **Fatemeh Amir‐Rad:** validation, writing – original draft. **Momen Atieh:** data curation, investigation, formal analysis, writing – review and editing.

## Funding

The authors have nothing to report.

## Ethics Statement

Ethical approval was not required.

## Conflicts of Interest

The authors declare no conflicts of interest.

## Supporting information


**Table S1:** Excluded studies after full‐text review.
**Table S2:** CBCT–DXA Methodological and Imaging Characteristics of Included Studies.
**Table S3:** Diagnostic performance of CBCT compared with DXA.

## Data Availability

The data that support the findings of this study are available on request from the corresponding author. The data are not publicly available due to privacy or ethical restrictions.

## References

[cre270423-bib-0001] Anderson, P. A. , S. L. Morgan , D. Krueger , et al. 2019. “Use of Bone Health Evaluation in Orthopedic Surgery: 2019 ISCD Official Position.” Journal of Clinical Densitometry 22, no. 4: 517–543. 10.1016/j.jocd.2019.07.013. 31519473

[cre270423-bib-0002] Barngkgei, I. , I. Al Haffar , and R. Khattab . 2014. “Osteoporosis Prediction From the Mandible Using Cone‐Beam Computed Tomography.” Imaging Science in Dentistry 44, no. 4: 263–271. 10.5624/isd.2014.44.4.263. 25473633 PMC4245467

[cre270423-bib-0003] Barngkgei, I. , I. Al Haffar , E. Shaarani , R. Khattab , and A. Mashlah . 2016. “Assessment of Jawbone Trabecular Bone Structure Amongst Osteoporotic Women by Cone‐Beam Computed Tomography: The OSTEOSYR Project.” Journal of Investigative and Clinical Dentistry 7, no. 4: 332–340. 10.1111/jicd.12170. 26097193

[cre270423-bib-0004] Barngkgei, I. , E. Joury , and A. Jawad . 2015. “An Innovative Approach in Osteoporosis Opportunistic Screening by the Dental Practitioner: The Use of Cervical Vertebrae and Cone Beam Computed Tomography With Its Viewer Program.” Oral Surgery, Oral Medicine, Oral Pathology and Oral Radiology 120, no. 5: 651–659. 10.1016/j.oooo.2015.08.008. 26453386

[cre270423-bib-0005] Barra, S. G. , J. A. A. de Arruda , A. F. Souza , et al. 2022. “Indices in Dental Image Exams for Bone Mineral Density Evaluation of Aromatase Inhibitor Users.” Brazilian Oral Research 36: e138. 10.1590/1807-3107bor-2022.vol36.0138. 36477215

[cre270423-bib-0006] Barra, S. G. , I. P. Gomes , T. M. P. Amaral , C. B. Brasileiro , L. G. Abreu , and R. A. Mesquita . 2021. “New Mandibular Indices in Cone Beam Computed Tomography to Identify Low Bone Mineral Density in Postmenopausal Women.” Oral Surgery, Oral Medicine, Oral Pathology and Oral Radiology 131, no. 3: 347–355. 10.1016/j.oooo.2020.07.016. 32843313

[cre270423-bib-0007] Boutroy, S. , M. L. Bouxsein , F. Munoz , and P. D. Delmas . 2005. “In Vivo Assessment of Trabecular Bone Microarchitecture by High‐Resolution Peripheral Quantitative Computed Tomography.” Journal of Clinical Endocrinology & Metabolism 90, no. 12: 6508–6515. 10.1210/jc.2005-1258. 16189253

[cre270423-bib-0008] Burt, L. A. , P. M. Wyatt , A. Morrison , and S. K. Boyd . 2023. “Bone Quality in Competitive Athletes: A Systematic Review.” Journal of Musculoskeletal & Neuronal Interactions 23, no. 4: 456–470. 38037364 PMC10696374

[cre270423-bib-0009] Carvalho, B. F. , J. G. K. de Castro , N. S. de Melo , et al. 2022. “Fractal Dimension Analysis on CBCT Scans for Detecting Low Bone Mineral Density in Postmenopausal Women.” Imaging Science in Dentistry 52, no. 1: 53–60. 10.5624/isd.20210172. 35387102 PMC8967487

[cre270423-bib-0010] de Castro, J. G. K. , B. F. Carvalho , N. S. de Melo , et al. 2020. “A New Cone‐Beam Computed Tomography‐Driven Index for Osteoporosis Prediction.” Clinical Oral Investigations 24, no. 9: 3193–3202. 10.1007/s00784-019-03193-4. 31912243

[cre270423-bib-0011] Cosman, F. , S. J. de Beur , M. S. LeBoff , et al. 2014. “Clinician's Guide to Prevention and Treatment of Osteoporosis.” Osteoporosis International 25, no. 10: 2359–2381. 10.1007/s00198-014-2794-2. 25182228 PMC4176573

[cre270423-bib-0012] Deeb, G. , L. Antonos , S. Tack , C. Carrico , D. Laskin , and J. G. Deeb . 2017. “Is Cone‐Beam Computed Tomography Always Necessary for Dental Implant Placement?” Journal of Oral and Maxillofacial Surgery 75, no. 2: 285–289. 10.1016/j.joms.2016.11.005. 27912075

[cre270423-bib-0013] Esmaeli, F. , S. Payahoo , M. Mobasseri , M. Johari , and J. Yazdani . 2017. “Efficacy of Radiographic Density Values of the First and Second Cervical Vertebrae Recorded by CBCT Technique to Identify Patients With Osteoporosis and Osteopenia.” Journal of Dental Research, Dental Clinics, Dental Prospects 11, no. 3: 189–194. 10.15171/joddd.2017.034. 29184636 PMC5666220

[cre270423-bib-0014] Fahd, A. , and D. ElBeshlawy . 2023. “Cone Beam Computed Tomography and Preoperative Bone Quality Assessment for Dental Implants: Myth and Truth.” ERU Research Journal 2: 541–549.

[cre270423-bib-0015] Francisco, I. , C. Nunes , F. Pereira , et al. 2023. “Bone Mineral Density Through DEXA and CBCT: A Systematic Review With Meta‐Analysis.” Applied Sciences 13: 5962. 10.3390/app13105962.

[cre270423-bib-0016] Gausden, E. B. , B. U. Nwachukwu , J. J. Schreiber , D. G. Lorich , and J. M. Lane . 2017. “Opportunistic Use of CT Imaging for Osteoporosis Screening and Bone Density Assessment: A Qualitative Systematic Review.” Journal of Bone and Joint Surgery 99, no. 18: 1580–1590. 10.2106/jbjs.16.00749. 28926388

[cre270423-bib-0017] Grados, F. , J. Fechtenbaum , E. Flipon , S. Kolta , C. Roux , and P. Fardellone . 2009. “Radiographic Methods for Evaluating Osteoporotic Vertebral Fractures.” Joint, Bone, Spine 76, no. 3: 241–247. 10.1016/j.jbspin.2008.07.017. 19196531

[cre270423-bib-0018] Guerra, E. N. S. , F. T. Almeida , F. V. Bezerra , et al. 2017. “Capability of CBCT to Identify Patients With Low Bone Mineral Density: A Systematic Review.” Dentomaxillofacial Radiology 46, no. 8: 20160475. 10.1259/dmfr.20160475. 28555506 PMC5965944

[cre270423-bib-0019] Güngör, E. , D. Yildirim , and R. Çevik . 2016. “Evaluation of Osteoporosis in Jaw Bones Using Cone Beam CT and Dual‐Energy X‐Ray Absorptiometry.” Journal of Oral Science 58, no. 2: 185–194. 10.2334/josnusd.15-0609. 27349539

[cre270423-bib-0020] Johnell, O. , and J. A. Kanis . 2006. “An Estimate of the Worldwide Prevalence and Disability Associated With Osteoporotic Fractures.” Osteoporosis International 17, no. 12: 1726–1733. 10.1007/s00198-006-0172-4. 16983459

[cre270423-bib-0021] Kato, C. N. , N. P. Tavares , S. G. Barra , et al. 2019. “Digital Panoramic Radiography and Cone‐Beam CT as Ancillary Tools to Detect Low Bone Mineral Density in Post‐Menopausal Women.” Dentomaxillofacial Radiology 48, no. 2: 20180254. 10.1259/dmfr.20180254. 30306800 PMC6476379

[cre270423-bib-0022] Kennel, K. A. , J. G. Sfeir , and M. T. Drake . 2020. “Optimizing DXA to Assess Skeletal Health: Key Concepts for Clinicians.” Journal of Clinical Endocrinology & Metabolism 105, no. 12: 3784–3791. 10.1210/clinem/dgaa632. 32894765

[cre270423-bib-0023] McInnes, M. D. F. , D. Moher , B. D. Thombs , et al. 2018. “Preferred Reporting Items for a Systematic Review and Meta‐Analysis of Diagnostic Test Accuracy Studies: The PRISMA‐DTA Statement.” Journal of the American Medical Association 319, no. 4: 388–396. 10.1001/jama.2017.19163. 29362800

[cre270423-bib-0024] Miller, S. A. , and J. L. Forrest . 2001. “Enhancing Your Practice Through Evidence‐Based Decision Making: Pico, Learning How to Ask Good Questions.” Journal of Evidence Based Dental Practice 1, no. 2: 136–141. 10.1016/S1532-3382(01)70024-3.

[cre270423-bib-0025] Page, M. J. , J. E. McKenzie , P. M. Bossuyt , et al. 2021. “The PRISMA 2020 Statement: An Updated Guideline for Reporting Systematic Reviews.” BMJ 372: n71. 10.1136/bmj.n71. 33782057 PMC8005924

[cre270423-bib-0026] Poiana, I. R. , R. Dobre , R. I. Popescu , S. M. Pituru , and A. Bucur . 2023. “Utility of Cone‐Beam Computed Tomography in the Detection of Low Bone Mass‐A Systematic Review.” Journal of Clinical Medicine 12, no. 18: 5890. 10.3390/jcm12185890. 37762831 PMC10531931

[cre270423-bib-0027] Professor Kanis, J. A. , L. J. Melton , C. Christiansen , C. C. Johnston , and N. Khaltaev . 1994. “The Diagnosis of Osteoporosis.” Journal of Bone and Mineral Research 9, no. 8: 1137–1141. 10.1002/jbmr.5650090802. 7976495

[cre270423-bib-0028] Reginster, J. Y. , and N. Burlet . 2006. “Osteoporosis: A Still Increasing Prevalence.” Bone 38, no. 2 Sl 1: 4–9. 10.1016/j.bone.2005.11.024. 16455317

[cre270423-bib-0029] Sennerby, L. , P. Andersson , L. Pagliani , et al. 2015. “Evaluation of a Novel Cone Beam Computed Tomography Scanner for Bone Density Examinations in Preoperative 3D Reconstructions and Correlation With Primary Implant Stability.” Clinical Implant Dentistry and Related Research 17, no. 5: 844–853. 10.1111/cid.12193. 24373386

[cre270423-bib-0030] Shah, A. 2017. “Implications of CBCT in Dentistry‐A Review.” Medical & Clinical Reviews 3, no. 3: 15. 10.21767/2471-299X.1000057.

[cre270423-bib-0031] Shokri, A. , M. Ghanbari , F. H. Maleki , L. Ramezani , P. Amini , and L. Tapak . 2019. “Relationship of Gray Values in Cone Beam Computed Tomography and Bone Mineral Density Obtained by Dual Energy X‐Ray Absorptiometry.” Oral Surgery, Oral Medicine, Oral Pathology and Oral Radiology 128, no. 3: 319–331. 10.1016/j.oooo.2019.04.017. 31171482

[cre270423-bib-0032] Slaidina, A. , E. Nikitina , A. Abeltins , U. Soboleva , and A. Lejnieks . 2022. “Gray Values of the Cervical Vertebrae Detected by Cone Beam Computed Tomography for the Identification of Osteoporosis and Osteopenia in Postmenopausal Women.” Oral Surgery, Oral Medicine, Oral Pathology and Oral Radiology 133, no. 1: 100–109. 10.1016/j.oooo.2021.06.014. 34535433

[cre270423-bib-0033] Slaidina, A. , B. Springe , A. Abeltins , S. E. Uribe , and A. Lejnieks . 2023. “The Effect of General Bone Mineral Density on the Quantity and Quality of the Edentulous Mandible: A Cross‐Sectional Clinical Study.” Dentistry Journal 11, no. 1: 17. 10.3390/dj11010017. 36661554 PMC9858291

[cre270423-bib-0034] Vasikaran, S. , R. Eastell , O. Bruyère , et al. 2011. “Markers of Bone Turnover for the Prediction of Fracture Risk and Monitoring of Osteoporosis Treatment: A Need for International Reference Standards.” Osteoporosis International 22, no. 2: 391–420. 10.1007/s00198-010-1501-1. 21184054

