# Supplementary material for: Comparative Diagnostic Performance of Cone Beam Computed Tomography and Dual‐Energy X‐Ray Absorptiometry for Low Bone Density Assessment. A Systematic Review and Meta‐Analysis
Source: Clin Exp Dent Res. 2026 Aug 1;12(4):e70423. doi: 10.1002/cre2.70423 (PMC13428463; doi:10.1002/cre2.70423)
Supplement: Supplementary file 1 — Table S1: Excluded studies after full‐text review. Table S2: CBCT–DXA Methodological and Imaging Characteristics of Included Studies. Table S3: Diagnostic performance of CBCT compared with DXA. [file CRE2-12-e70423-s001.docx]

**Supplementary Material**

**Table S1. Excluded studies after full-text review**

| Study ID | PMID | Reason for exclusion |
| --- | --- | --- |
| Sghaireen, et al 2023 | 37048761 | Lack of diagnostic performance metrices |
| Abdinian, et al 2023 | 37308822 | Lack of diagnostic performance metrices |
| Brasileiro, et al 2017 | 28265896 | Lack of diagnostic performance metrices |
| Koh et al., 2011 | 22010066 | Lack of diagnostic performance metrices |
| Mostafa et al., 2016 | 27418348 | Lack of diagnostic performance metrices |
| Albayati et al, 2018 | 30210186 | Lack of diagnostic performance metrices |
| Ko et al, 2016 | 27588253 | Artifact-based BMD estimation no DXA comparison |
| Liu et al., 2017 | 28125517 | Focuses on bone quality for implants, no osteoporosis diagnosis or DXA comparison |
| Geibel, et al 2016 | 27681530 | Full text in German |
| Blake, et al 2005 | 15645160 | Evaluates Con Beam DXA without comparison with CBCT |

**Table S2. CBCT–DXA Methodological and Imaging Characteristics of Included Studies.**

| **Study ID** | **DXA device** | **DXA exposure** | **CBCT device** | **CBCT exposure parameters** | **FOV** | **Voxel / slice thickness (mm)** | **Observer(s)** | **Blinding to DXA** | **Analysis software** | **BMD / CBCT parameter** |
| --- | --- | --- | --- | --- | --- | --- | --- | --- | --- | --- |
| Slaidina et al., 2021 | Lunar DPX-NT (GE) | NR | i-CAT Next Generation | 120 kVp, 5 mA, 4 s | 230 × 115 mm | 0.30 | 1 dentist (15 yrs), read twice | NR | OnDemand3D | Gray values |
| Slaidina et al., 2023 | NR | NR | i-CAT Next Generation | 120 kVp, 5 mA, 4 s | 230 × 115 mm | 0.30 | 1 dentist (15 yrs), read twice | NR | OnDemand3D | Gray values |
| Barngkgei et al., 2014 | Hologic Discovery QDR | NR | WhiteFox | NR | 13 × 15 cm | 0.25 | NR | NR | WhiteFox Imaging v3 | Gray values |
| Barngkgei et al., 2015 | Hologic Discovery QDR | NR | WhiteFox | 105 kV, 9 mA, 9 s | 13 × 15 cm | 0.25 | NR | NR | WhiteFox Imaging v3 | Gray values |
| Esmaeili et al., 2017 | Hologic QDR 4500 | NR | NewTom VGi | 110 kVp, 1–20 mA, 18 s | 15 × 12 cm | 0.20 | NR | NR | NNT NewTom | Gray values |
| Shokri et al., 2019 | Osteocore (Medilink) | 86 kVp, 9 mA | Scanora 3D | 90 kVp, 10 mA, 12.4 s | 13 × 14 cm | 0.50 | 2 observers | NR | OnDemand3D | Gray values |
| Barra et al., 2021 | Hologic Discovery DXA | NR | Carestream 9000C 3D | 72 kVp, 10 mA, 32.4 s | 50 × 37 mm | 0.076 | 1 trained examiner | Yes | Implant Viewer 3.5 | Radiomorphometric indices |
| Barra et al., 2022 | Hologic Discovery | NR | Carestream 9000C 3D | 72 kVp, 10 mA, 32.4 s | 50 × 37 mm | 0.20 | 1 radiologist | NR | Imaging Studio 3.2 | Radiomorphometric indices |
| Kato et al., 2019 | Hologic Discovery DXA | NR | Carestream 9000C 3D | 72 kVp, 10 mA, 32.4 s | 50 × 37 mm | 0.20 | 2 radiologists | Yes | Imaging Studio 3.2 | Mandibular cortical index |
| de Castro et al., 2020 | Lunar DPX-NT (GE) | NR | i-CAT Classic | 120 kVp, 8 mA, 40 s | 8 × 8 cm | 0.25 | 2 radiologists | Yes | ImageJ 1.47 | 3D mandibular osteoporotic indices |
| Carvalho et al., 2022 | Lunar DPX-NT (GE) | NR | i-CAT Classic | 120 kVp, 8 mA, 40 s | 20 × 8 cm | 0.25 / 1.25 | 2 radiologists | Yes | Xoran + BoneJ | Fractal dimension |
| Güngör et al., 2016 | Hologic Discovery QDR | NR | i-CAT Vision | 120 kVp, 18.59 mA, 8.9 s | 130 × 100 mm | 0.21 | NR | NR | i-CAT Vision, ImageJ | FD, indices, gray values |

NR= Not Reported, FOV= Field of View, kVp=kilovolt peak, mA= milliamperes, BMD= Bone Mineral Density

**Table S3. Diagnostic performance of CBCT compared with DXA.**

| **Study ID** | **CBCT site** | **Sensitivity (%)** | **Specificity (%)** | **Accuracy (%)** | **p-value** |
| --- | --- | --- | --- | --- | --- |
| Slaidina et al., 2021 | C2 axial gray value | 45.5 | 87.2 | 58.3 | NR |
|  | C2 sagittal gray value | 61.4 | 69.2 | 63.8 | NR |
|  | C3 axial gray value | 44.8 | 87.2 | 57.9 | NR |
|  | C3 sagittal gray value | 63.2 | 59.0 | 61.9 | NR |
| Slaidina et al., 2023 | Lateral incisor CM (gray value) | 98.9 | 100.0 | 99.2 | NR |
|  | Lateral incisor trabecular basal | 81.8 | 79.5 | 81.1 | NR |
|  | Lateral incisor trabecular alveolar | 81.8 | 79.5 | 81.1 | NR |
|  | First premolar CM (gray value) | 98.9 | 100.0 | 99.2 | NR |
|  | First premolar trabecular basal | 72.7 | 74.4 | 73.2 | NR |
|  | First premolar trabecular alveolar | 72.7 | 74.4 | 73.2 | NR |
|  | First molar CM (gray value) | 100.0 | 100.0 | 100.0 | NR |
|  | First molar trabecular basal | 51.1 | 48.7 | 50.4 | NR |
|  | First molar trabecular alveolar | 51.1 | 48.7 | 50.4 | NR |
| Barngkgei et al., 2014 | Mandibular body slice (gray value) | 46.4 | 90.0 | 57.9 | NR |
|  | Mandibular body slice (gray value) | 50.0 | 88.2 | 67.6 | NR |
| Barngkgei et al., 2015 | Right C1 vertebral GV | 92.9 | 90.0 | 92.1 | NR |
|  | Left C1 vertebral GV | 92.9 | 80.0 | 89.5 | NR |
|  | C2 vertebral GV | 89.3 | 90.0 | 89.5 | NR |
|  | C2 dens GV | 71.4 | 90.0 | 76.3 | NR |
|  | Right C1 vertebral GV | 81.0 | 70.6 | 76.3 | NR |
|  | Left C1 vertebral GV | 61.9 | 94.1 | 76.3 | NR |
|  | C2 vertebral GV | 71.4 | 82.4 | 76.3 | NR |
|  | C2 dens GV | 76.2 | 76.5 | 76.3 | NR |
| Esmaeili et al., 2017 | Left lateral mass of C1 (gray value) | 86.2 | 100.0 | 92.6 | NR |
|  | Right lateral mass of C1 (gray value) | 91.4 | 100.0 | 95.4 | NR |
|  | Body of C2 (gray value) | 93.1 | 100.0 | 96.3 | NR |
|  | Dens of C2 (gray value) | 70.7 | 68.0 | 69.4 | NR |
| Shokri et al., 2019 | Mandibular incisors ROI (cancellous+cortical GV) | 61.8 | 44.4 | 54.1 | NR |
|  | Mandibular premolars ROI (cancellous+cortical GV) | 55.9 | 44.4 | 50.8 | NR |
|  | Mandibular retromolar ROI (cancellous+cortical GV) | 61.8 | 51.9 | 57.4 | NR |
|  | Maxillary incisors ROI (cancellous+cortical GV) | 47.1 | 51.9 | 49.2 | NR |
|  | Maxillary premolars ROI (cancellous+cortical GV) | 52.9 | 48.1 | 50.8 | NR |
|  | Maxillary tuberosity ROI (cancellous+cortical GV) | 58.8 | 59.3 | 59.0 | NR |
| Barra et al., 2022 | Posterior (P) index | 34.4 | 87.5 | 45.0 | NR |
|  | Molar (M) index | 37.5 | 87.5 | 47.5 | NR |
|  | Anterior (A) index | 46.9 | 75.0 | 52.5 | NR |
|  | Symphysis (S) index | 84.4 | 37.5 | 75.0 | NR |
|  | CT Mandibular Index (CTMI) | 9.4 | 62.5 | 20.0 | NR |
|  | CT Index inferior [CTI (I)] | 71.9 | 50.0 | 67.5 | NR |
|  | CT Index superior [CTI (S)] | 56.2 | 62.5 | 57.5 | NR |
| Barra et al., 2021 | Molar (M) index | 75.0 | 68.8 | 72.9 | NR |
|  | Symphysis (S) index | 50.0 | 87.5 | 62.5 | NR |
|  | Posterior (P) index | 75.0 | 62.5 | 70.8 | NR |
| Kato et al., 2019 | Panoramic recon 5 mm (radiomorphometric index) | 63.2 | 43.8 | 57.4 | NR |
|  | Panoramic recon 15 mm (radiomorphometric index) | 50.0 | 50.0 | 50.0 | NR |
|  | Panoramic recon 25 mm (radiomorphometric index) | 52.6 | 62.5 | 55.6 | NR |
| Barngkgei et al., 2016 | Trabecular thickness (Tb.Th) of dens | 61.9 | 88.2 | 73.7 | NR |
|  | Maximum Tb.Th of dens (mm) | 19.0 | 94.1 | 52.6 | NR |
|  | Trabecular thickness (Tb.Th) of dens | 60.7 | 80.0 | 65.8 | NR |
|  | Maximum Tb.Th of dens (mm) | 39.3 | 90.0 | 52.6 | NR |
| de Castro et al., 2020 | 3D-MOI CQ | 54.9 | 92.3 | 73.8 | NR |
|  | 3D-MOI PR (2.75 mm) | 78.4 | 67.3 | 72.8 | NR |
|  | 3D-MOI CS (2.75 mm) | 76.5 | 69.2 | 72.8 | NR |
| Carvalho et al., 2022 | Mandibular ROI-m (fractal dimension) | 54.9 | 71.2 | 63.1 | NR |
| Güngör et al., 2016 | Right maxilla (fractal dimension) | 91.5 | 93.5 | 92.2 | NR |
|  | Left maxilla (fractal dimension) | 91.5 | 96.8 | 93.3 | NR |
|  | Right condyle (fractal dimension) | 83.1 | 87.1 | 84.4 | NR |
|  | Left condyle (fractal dimension) | 89.8 | 90.3 | 90.0 | NR |
|  | CTMI (radiomorphometric index) | 93.2 | 83.9 | 90.0 | NR |
|  | CTI (I) (radiomorphometric index) | 94.9 | 90.3 | 93.3 | NR |
|  | CTI (S) (radiomorphometric index) | 94.9 | 90.3 | 93.3 | NR |
|  | Right maxilla (gray value) | 72.9 | 90.3 | 78.9 | NR |
|  | Left maxilla (gray value) | 79.7 | 83.9 | 81.1 | NR |
|  | Right mandible (gray value) | 84.7 | 83.9 | 84.4 | NR |
|  | Left mandible (gray value) | 91.5 | 80.6 | 87.8 | NR |
|  | Right condyle (gray value) | 30.5 | 45.2 | 35.6 | NR |
|  | Left condyle (gray value) | 72.9 | 83.9 | 76.7 | NR |

NR= not reported, s= seconds, kv= Kilo Voltage, mA= milliamperes, P: Posterior, A: Anterior, M: Molar, PM: Premolar, S: Symphysis, CTMI: Cortical Thickness Mental Index, CTI (S): Cortical Thickness Index (Superior), CTI (I)Cortical Thickness Index (Inferior)
